# AI and Technology Enabled Clinical Workflow Redesign

**DOI:** 10.1089/tmr.2024.0079

**Published:** 2024-12-23

**Authors:** Lee H. Schwamm, Sarah Pletcher, Alistair Erskine

**Affiliations:** ^1^ Yale School of Medicine, New Haven, Connecticut, USA.; ^2^ Houston Methodist/Weill Cornell Medicine, Houston, Texas, USA.; ^3^ Emory University, Atlanta, Georgia, USA.

**Keywords:** ambient documentation, sustainability, telenursing, virtual care

## Abstract

The rapid evolution of digital health technologies has significantly transformed health care delivery, particularly in the realm of virtual care. This article synthesizes insights from the Annual Virtual Care Symposium entitled “Virtual Care: Embedding Virtual Care into Post-Pandemic Healthcare Delivery” held on March 13, 2024, hosted by Yale School of Medicine, Emory University, University of Washington, and Mayo Clinic Arizona. By examining the initiatives presented by faculty from Yale, Houston Methodist, and Emory in this session, we explore the integration of advanced technologies in virtual care, the challenges faced, and the potential for improving patient outcomes and health care efficiency as these novel practices become systematized and sustainable. The session focused on artificial intelligence and technology-enabled clinical workflow redesign.

## Introduction

The COVID-19 pandemic has accelerated the adoption of virtual care, highlighting the need for innovative solutions to enhance health care delivery. This article reviews recent advancements, focusing on the integration of advanced technologies in virtual care. We aim to provide a comprehensive overview of these initiatives, their impact on health care, and the lessons learned.

### Methods

This article is a summary of the proceedings of a virtual symposium. No human research was performed, and therefore no IRB approval was required.

### Symposium content

Dr. Schwamm presented an overview of virtual care and artificial intelligence (AI). Virtual care has proven to be an effective tool in maintaining health care services during the pandemic,^1,2^ though payment and coverage parity remain uncertain at the state and federal level. The challenge now is to integrate virtual care into the routine health care delivery model. This involves addressing several key areas, including clinical workflow redesign, assessing clinical appropriateness, technology and IT infrastructure, and patient preference/engagement. Virtual care requires a redesign of clinical workflows to ensure seamless integration with traditional in-person care. This includes training health care providers to use virtual care tools effectively and redesigning patient pathways to incorporate virtual consultations.^3^ Robust IT infrastructure is essential for the successful implementation of virtual care. This includes secure and reliable internet connections, telehealth platforms, and electronic health records (EHRs) systems that support virtual consultations. In addition, engaging patients in virtual care is crucial for its success. This involves educating patients about the benefits of virtual care, providing user-friendly platforms, and ensuring that virtual care services are accessible to all patients, including those with limited digital literacy.^4^ Lastly, more randomized controlled trials are needed to establish the evidence of benefits for virtual versus traditional care delivery models since the pandemic social isolation measures accelerated adoption by necessity ahead of the accumulation of high-quality data.^5^

AI and technology play a pivotal role in redesigning clinical workflows and improving patient care. AI can automate repetitive tasks, provide decision support, and enhance the accuracy of diagnoses. To better understand this, we need to further classify AI into different subcategories of robotic process automation (RPA), machine learning (ML), and the newest entrant of generative AI (GenAI) in escalating order of complexity and computational demand.

RPA involves software robots that automate repetitive, rule-based tasks such as data entry and invoice processing. This reduces the administrative burden on health care providers and allows them to focus on patient care. ML enables systems to learn from data and make predictions or decisions without being explicitly programmed. In health care, ML can be used for predictive analytics, speech recognition, and personalized treatment plans. Lastly, GenAI can create new content, such as clinical documentation, reducing the cognitive burden on health care providers. It can also generate personalized educational content for patients, improving patient engagement and adherence to treatment plans.

GenAI also has the potential to revolutionize clinical documentation by automating the creation of clinical notes and other documentation tasks. This reduces the cognitive burden on health care providers and allows them to spend more time with patients. The two most prevalent examples of widely adopted GenAI in clinical medicine are ambient listening for clinical documentation and automated reply technology to draft responses to patient portal messages for clinicians. GenAI-Powered Ambient Listening can listen to clinician–patient conversations and automatically generate clinical notes. This reduces the time health care providers spend on documentation and increases the time they can spend with patients.^6^ This technology is spreading rapidly across health systems and gaining traction as a pragmatic solution to one aspect of clinician burnout due to administrative burden, a problem facing all of health care. GenAI Drafts for Portal Messaging can generate first drafts of patient messages in provider portals, reducing the time health care providers spend on routine communication tasks.^7^ This improves efficiency and allows providers to focus on patient care and high-level decision-making.

### Data management: Secure and efficient data management is critical for leveraging AI in health care

Secure and efficient data management is vital for leveraging AI in health care. This involves several key components:

1. Data Storage and Access: Access to abundant and secure storage and high-performance computing infrastructure is essential for managing large volumes of health care data. This includes secure access to curated, multi-dimensional data collected in clinical and nonclinical settings.
2. Data Governance and Policies: Robust governance and policies on data use are crucial for ensuring data security and privacy. This includes methods for secure aggregation and deidentification of structured and unstructured data.
3. Standard Data Models: The use of standard data models, such as The Observational Medical Outcomes Partnership (OMOP) Common Data Model (CDM), is an open community data standard, designed to standardize the structure and content of observational data and to enable efficient analyses that can produce reliable evidence. A central component of the OMOP CDM is the use of standardized vocabularies. These standard vocabularies allow organization and standardization of medical terms to be used across the various clinical domains of the OMOP common data model and enable standardized analytics that leverage the knowledge base when constructing exposure and outcome phenotypes and other features within characterization, population-level effect estimation, and patient-level prediction studies. It also supports the concept of federated queries and collaboration across different health care entities, including payors, academic medical centers, and industry partners.^8^

### Key requirements for successful AI adoption in health care

In addition to the components mentioned above, successful AI adoption in health care requires addressing model transparency and explainability, the nature of human-AI collaboration, the regulatory framework in which this will all sit, and the workforce training and education that will be necessary. AI systems must be transparent and explainable to ensure trust and acceptance by health care providers and patients. This involves providing clear explanations of how AI algorithms make decisions, what training data sets were used, and how diverse and free from bias these training data sets.^9^ Effective collaboration between humans and AI systems is crucial for maximizing the benefits of AI in health care. This involves training health care providers to work with AI tools and ensuring that AI systems complement, rather than replace, human expertise. Workforce trust is essential for the safe and responsible adoption of such rapidly evolving technology, one that has the potential to reshape the workforce and shed certain layers of the workforce and replace them with more agile knowledge workers. An evolving regulatory framework is necessary to ensure the safe and effective use of AI in health care. This includes guidelines for AI development, pre-deployment validation, purchaser evaluation of AI systems with local validation, and post-deployment monitoring of these systems to ensure the promised performance is delivered and that results do not discriminate between different patient groups or drift over time. Lastly, training and education are essential for preparing health care providers to use AI tools effectively. This includes providing ongoing education and support to ensure that providers are comfortable and proficient in using AI systems.

## A Deep Dive into AI Ambient Listening at Emory: Enhancing Patient and Provider Experience

Dr. Erskine presented on the *Ambient Listening Program at Emory*, an innovative initiative aimed at leveraging AI technology to improve the documentation process in health care settings. This program focuses on using AI ambient listening technology to record patient conversations via a mobile app, which then generates digital notes that can be reviewed, edited, and finalized within the EHR system, specifically Epic.

### Impact on experience and wellness

The digital notes created through this program have a significant impact on both patient and provider experience and wellness. For patients, the use of AI-generated notes ensures that their conversations with health care providers are accurately documented, leading to better continuity of care. For providers, this technology reduces the administrative burden associated with manual notetaking, allowing them to focus more on patient care. The providers also benefit from reduced cognitive load, as the burden on working memory is diminished by AI-generated recording of events and follow-up tasks. The implementation of digital notes has led to a noticeable improvement in the overall experience and wellness of both patients and providers.

### Ease of implementation and expansion

The solution chosen by Emory offers an out-of-the-box implementation that includes Software-as-a-Service (SaaS), native EHR integration, and tailored support. The SaaS model ensures that the software is cloud-based, eliminating the need for local installations and reducing the associated effort. The enterprise licensure model accommodates many potential users, as seen in Emory’s case with approximately 9,000 established use case roles (2,500 attendings, 1,000 APP/residents, and 5,000 nurses). This can be expanded to other users who might include care managers, social workers, and pharmacist, but the AI-generated note templates are not yet tailored to these expanded users. The native integration with Epic enables rich data collection and prevents the need for custom integration work. In addition, the vendor’s tailored roadmap ensures that the company can effectively accommodate support requests and provide timely assistance.

The program has seen rapid expansion and high adoption rates. Initially, the program started with 16 providers across seven specialties. Within a few months, it expanded to include 429 providers across multiple specialties and currently exceeds 1,900 providers. This rapid growth demonstrates the scalability and effectiveness of the Ambient Listening Program. The high adoption and retention rates observed were associated with significant productivity improvements. For instance, among the top 25% of adopters with full performance data, there was a notable increase of 7% in the number of appointments closed on the same day and an increase of 0.33 additional appointments held per day. Data presented on experience included a provider Net Promoter Score of 13.5 (*n* = 55 providers surveyed) with post-implementation improvements of 32% in usability, 13% in patient experience, and 40% in provider wellness. Adoption was achieved in 78% of those trying the product, and retention occurred in 82% of those activated.

The emerging results from the program indicate high adoption and retention rates, along with significant productivity improvements. The program has seen a 78% activation rate and an 82% retention rate among providers. In addition, there has been a 7% increase in the number of appointments closed on the same day and a 0.33 increase in the number of appointments held per day among the top adopters. Looking ahead, the program aims to increase adoption and scope further. Plans include the development of an adoption dashboard to show productivity potential and user opportunities. The app integration of the software with Haiku, a mobile app for Epic, is also in development, allowing for native mobile enrollment and obviating the need for separate user accounts, identity and access management, and data security. Furthermore, the program is piloting expanded use in inpatient and emergency department settings through a standalone workflow.

### Lessons learned and considerations for adoption

Several lessons learned and considerations for adoption should be considered. Some of the rate-limiting factors encountered include distrust of AI, lack of familiarity with Epic, and limitations of third-party apps and web portals. In addition, some providers expressed concerns about the AI not generating specialty-specific output, and there were challenges related to Android limitations and the belief that using the technology might lead to increased patient loads. Despite these challenges, the feedback from providers has been largely positive. Addressing concerns early and providing adequate support to clinicians is vital to ensure successful adoption and implementation of the AI ambient listening technology.

## Tech-Enabled Care Redesign: A Key Element to Building a Smart Hospital

Dr. Pletcher from Houston Methodist shared insights into their efforts to leverage technology to redesign care delivery, with a focus on improving patient outcomes and clinician experiences. Leading a broad initiative of care redesign/transformation programs, including a “Smart Hospital technology portfolio,” Dr. Pletcher presented Houston Methodist’s vision to integrate advanced technologies into health care practices, ensuring a seamless and efficient care delivery system.

Houston Methodist is investing in a range of innovations to create this future-focused hospital model, including a wide range of virtual service, patient self-scheduling, smart rooms, ambient intelligence, service robotics, remote monitoring, integrated clinical and financial systems, multi-modal education, and predictive and proactive AI. Together, these innovations aim to improve patient care and operational efficiency, creating a responsive health care environment that adapts to patient and provider needs in real time.

### TeleNursing

A cornerstone of this tech-enabled redesign is TeleNursing, which provides remote support for bedside operations and enables personalized patient care while reducing the burden on bedside clinicians. TeleNursing tasks range from completing admission questionnaires and documenting nursing admission profiles in the EMR to placing needed consults, reviewing discharge instructions, confirming pharmacy orders, and providing pre-op and inpatient support. Equipped with technology including wall-mounted A/V room systems, carts, tablets, and secure messaging, TeleNursing has scaled rapidly, supporting over 100,000 admissions and discharges, with an average call duration of 14 min and over 23,000 encounters in Q4-2023. Expanded use cases also include rounding to assess DVT prophylaxis treatment for which >100 rounding calls have been conducted.

### Care redesign impact

The care redesign efforts have led to meaningful improvements across multiple domains. Eliminating the need for contract labor has contributed to higher nursing satisfaction and retention. In addition, decreased telemetry use has freed up time for direct patient care activities, and a redesigned approach to bedside vitals assessment that leverages a wearable vitals button device has automated hourly vitals collection, reducing routine manual vitals checks from every 4 h to every 8 h, and even longer at night for stable patients. This automation has provided more time for bedside teams to focus on direct patient care and, through centralized monitoring of all hospitalized patients, has supported earlier detection of patient deterioration, enabling proactive interventions. Patients benefit from fewer sleep interruptions, while the new trending alerts have ensured equal or better safety levels compared to the previous manual model. In Q4-FY23, over 38,000 vital sign alerts were triggered for review by the telemetry team, of which 19,000 were sent to the virtual nurse to review, of which only 5,000 (∼13% of all alerts) were escalated for bedside evaluation.

A comprehensive telemetry redesign is underway, involving workflow review and redesign, AI-enabled algorithms and analytics, and full system centralization. The shift optimizes real-time telemetry monitoring, reducing alarm fatigue of floor-based staff and enhancing patient care. In addition, the transition from traditional 1:1 bedside sitting to a cart-based virtual sitting model, with mounted sensors with system integration, has made patient monitoring more dynamic, consistent, and cost-effective. In sum, nursing care redesign has positively impacted hospital performance, patient satisfaction, and clinical outcomes.

### Connected care and the quadruple aim

This vision for connected care aligns with the Quadruple Aim: better outcomes, improved patient experience and access, improved clinician experience, and lower costs. The connected care model aims to standardize care, improve access and compliance, enhance follow-up and metrics, and provide fast, convenient, and cost-effective care. It also focuses on remote and at-home care, continuity of care, and multidisciplinary care. For clinicians, the model aims to improve work-life balance, leverage their expertise effectively, decrease clerical workload, and enhance engagement. Cost savings stem from effective extender utilization, elimination of unnecessary tests and transfers, and improved recruitment and retention.

By leveraging innovations such as smart hospitals, TeleNursing, and connected care models, Houston Methodist aims to enhance patient outcomes, improve clinician experience, and reduce costs. The comprehensive care redesign efforts have already shown significant positive impacts on nursing satisfaction, patient care, and hospital performance. This transformation lays the groundwork for a sustainable, tech-enabled health care model poised to advance the future of healthcare delivery.

## Conclusion

The integration of advanced technologies in virtual care has the potential to transform health care delivery, improving patient outcomes and health care efficiency. The initiatives discussed in this article demonstrate the potential of speech recognition, natural language processing, tele-nursing, remote monitoring, RPA, and ML to enhance virtual care. However, the success of these initiatives depends on the seamless integration with existing clinical workflows, significant investment in infrastructure and training, and the ability to ensure the accuracy and quality of care provided.

By adopting a collaborative approach and leveraging the strengths of various health care facilities and professionals, we can overcome the challenges posed by the digital transformation in health care and enhance the delivery of virtual care. The lessons learned from these initiatives provide valuable insights for health care providers and policymakers as they navigate the evolving landscape of digital health.

## Abbreviations Used

APP: advanced practice provider
A/V: audiovisual
CDM: Common Data Model
DVT: deep vein thrombosis
EHR: electronic health record
Gen AI: generative artificial intelligence
IT: information technology
ML: machine learning
OMOP: Observational Medical Outcomes Partnership
RPA: robotic process automation
SaaS: Software-as-a-Service
